# Evolution of Recurrent Myxofibrosarcoma of the Thoracic Wall at Single-Cell Resolution: A Case Report

**DOI:** 10.3390/ijms27146229

**Published:** 2026-07-13

**Authors:** Elena E. Kopantseva, Artem L. Toropov, Timur I. Fetisov, Alexander V. Ikonnikov, Alexandra V. Sentyabreva, Maxim E. Menyailo, Anastasia A. Tararykova, Polina A. Shtompel, Victoria Y. Zinovieva, Kirill I. Kirsanov, Evgeny V. Denisov, Marianna G. Yakubovskaya

**Affiliations:** 1 Research Institute of Molecular and Cellular Medicine, Peoples’ Friendship University of Russia (RUDN University), 117198 Moscow, Russia; arteem2300@gmail.com (A.L.T.); timkatryam@yandex.ru (T.I.F.); alex.v.ikonnikov@gmail.com (A.V.I.); alexandraasentyabreva@gmail.com (A.V.S.); max1989me@gmail.com (M.E.M.); anastasiatararykova@gmail.com (A.A.T.); polina.shtooo@gmail.com (P.A.S.); kkirsanov85@yandex.ru (K.I.K.); mgyakubovskaya@mail.ru (M.G.Y.); 2 N.N. Blokhin National Medical Research Center of Oncology, 115478 Moscow, Russia; vichka2396@gmail.com; 3 Avtsyn Research Institute of Human Morphology, Petrovsky National Research Centre of Surgery, 119991 Moscow, Russia; 4 Cancer Research Institute, Tomsk National Research Medical Center, Russian Academy of Sciences, 634009 Tomsk, Russia

**Keywords:** myxofibrosarcoma, recurrence, metastasis, single-cell RNA sequencing, soft tissue sarcoma, transcriptome

## Abstract

Myxofibrosarcoma (MFS) is a common yet understudied type of soft tissue sarcoma. It is characterized by diverse cellular morphology, unusual growth patterns, and a propensity for local tumor recurrences. A 76-year-old patient underwent multiple surgical removals of MFS recurrences of the thoracic cavity and surrounding tissues with the subsequent development of metastases. Single-cell RNA sequencing was used to analyze the earlier (R7) and later (R8) MFS recurrences, with R8 removed less than a year before the detection of metastases in the lungs. The earlier MFS recurrence displays tumor clusters with multiple immunomodulatory markers (*DKK1*, *APP*, *CD24*, *GRN*) and genes involved in lipid metabolism and cell stress (*DDIT3*, *ABCA1*, *ABCA10*, *ATF4*, *NEU1*), combined with an anti-inflammatory TME. The later MFS recurrence shifts to a functionally less diverse phenotype, enriched in fibroblast-typical markers (*POSTN*, *NES*, *COL1A1/A2*), and a pro-inflammatory TME. The proliferative (*MKI67*, *TOP2A*, *BUB1B*) and mRNA splicing and processing (*SNRNP70*, *SRSF2/5/11*, *RSRP1*, *LUC7L/7L3*, *SRRM1/2*) tumor clusters are observed in both MFS recurrences, and their signatures match the previous data on primary MFS tumor populations. ScRNA-seq analysis of two subsequent MFS recurrences from one patient show a change from functionally diverse to more uniform ECM remodeling enriched tumor populations, an accompanying shift to the pro-inflammatory TME, and the presence of two common tumor clusters enriched in proliferative and mRNA-processing gene signatures.

## 1. Introduction

Myxofibrosarcoma (MFS) is a mesenchymal malignancy characterized by cell pleomorphism, myxoid stroma, and the presence of curvilinear vessels [1]. It is one of the most common and difficult-to-treat-subtypes of soft tissue sarcoma (STS). MFS constitutes about 5–20% of STS cases according to different sources [2,3,4] and mostly affects elderly patients. The main obstacles in its treatment are its poor response to chemotherapy and diverse growth patterns [5], which result in the high frequency (20–60%) of local recurrences and limb amputations (17–41%) and medium frequency (20–25%) of metastasis to the lungs and lymph nodes [3,6,7].

MFS demonstrates complexity and marked heterogeneity on the genomic and transcriptomic levels. Frequent DNA alterations in MFS include the TP53 gene, cell cycle checkpoint regulators (*CDKN2B*, *CCND1*, *CDKN2A*, and *CDK6*), and other genes involved in proliferation and development including *KRAS*, *WNT11*, *NTRK1*, *MDM2*, *GNAS*, *FOXA1*, *NKX2-1*, *SYK*, and *JAK1* [8]. Unique less frequent MFS subtypes display additional mutations in the cell cycle genes and the amplification of the *MDM2* gene [8]. Integrated analysis of genomic and transcriptomic data suggests the existence of several immune subtypes of MFS [8,9] as well as heterogeneous chemosensitivity within the group [10]. The transcriptomic landscape of primary MFS has been characterized at single-cell resolution [11], leading to the identification of functional groups of primary MFS tumor cells, their pathways of interaction with the tumor microenvironment (TME), and the differences between MFS and a highly similar sarcoma, the undifferentiated pleomorphic sarcoma (UPS). Overall, there is a need for integrated genomic and transcriptomic analysis, especially at the single-cell and spatial levels, to improve patient stratification and therapeutic decision-making for this highly heterogeneous STS group.

Less is known about the recurrent and metastatic MFS. A study of 536 STS transcriptomes reported that metastasis-related signatures in MFS are related to fatty acid degradation [12]. Low expression of CD34 and high expression of CD109, CD248, MET, and EZR have been linked to MFS metastasis [13,14,15,16]. However, the exact changes in the tumor and TME in MFS with multiple recurrences still need to be documented. The study of the temporal evolution of MFS is made additionally challenging by the intra-tumoral [17] and inter-tumoral [8] heterogeneity, with the possible solution requiring analysis of large patient cohorts with multiple tumor samples taken from each patient.

In this case report, we describe a patient with multiple MFS recurrences, which subsequently progress to metastases. Using single-cell RNA sequencing, we investigate the changes in tumor and TME cell populations between the two MFS recurrences and compare them to the previously acquired data on the primary MFS landscape [11]. We identify the transcriptional signatures that primary and recurrent MFS have in common and offer hypotheses about the mechanisms of MFS evolution over time.

## 2. Case Report

We present a case of a 76-year-old woman with a highly recurrent MFS (eight local recurrences) localized in the soft tissue of the thoracic cavity. Prior to being admitted to the N.N. Blokhin National Medical Research Center of Oncology (NMRCO), the patient underwent multiple surgical removals of the recurring MFS of the thoracic region (2013, 2014, 2015, 2017, and 2022). In 2015, after the surgical removal of the tumor in the anterior left part of the thoracic wall, a course of radiation therapy (single radiation dose—3 Gy, total radiation dose—54 Gy) was administered. In 2018, chemotherapeutic treatment with dacarbazine and doxorubicin was conducted after surgical intervention.

In 2022, the computer tomography (CT) of the thoracic cavity detected post-radiation treatment changes. The magnetic resonance imaging (MRI) did not show any pathological nodal growth. A recurrence at the original tumor site was suspected, and the puncture biopsy confirmed the presence of MFS cells. The surgical removal of the soft tissue tumor of the anterior part of the thoracic wall was conducted on 19 July 2022 (post-operation histology: MFS Grade 3). After the operation, the patient was dynamically monitored.

According to the positron emission tomography–computed tomography (PET/CT) conducted in June 2023, the subcutaneous fat layer in the inner lining of the upper third part of the left shoulder contained an enlargement of the soft tissue component. The biopsy confirmed a recurrence of MFS (R7). The surgical treatment with the exarticulation of the left shoulder was carried out on 14 August 2023 (post-operation histology: MFS Grade 3). After the operation, the patient was dynamically monitored.

**Figure 1:**
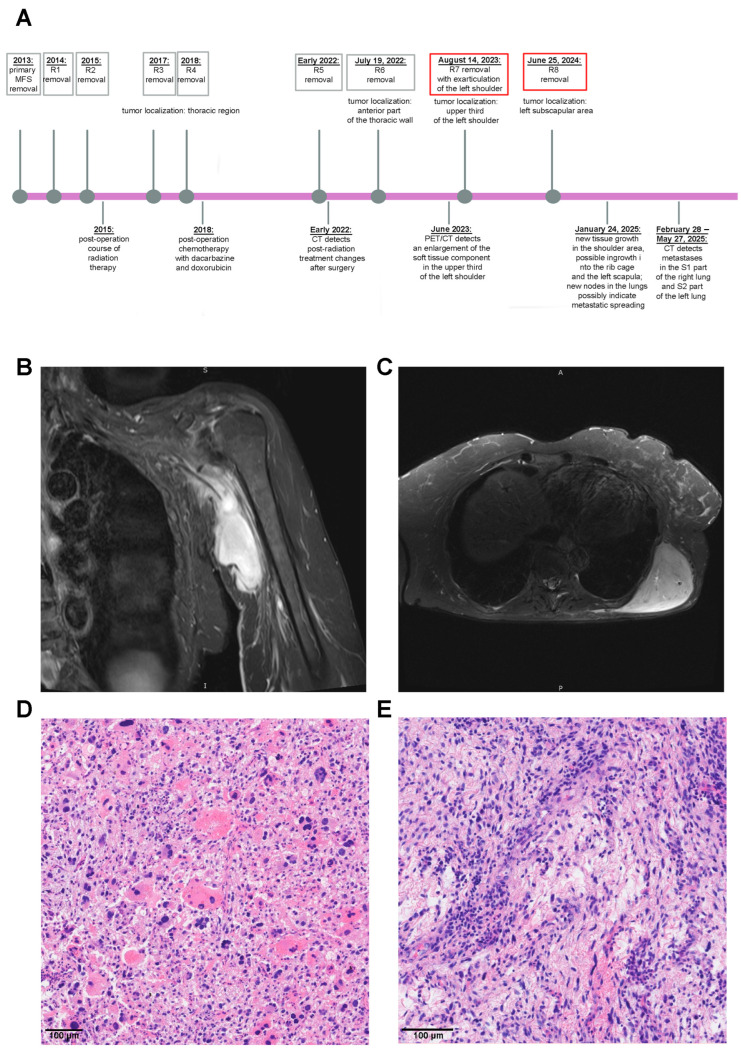
Clinical data on the R7 and R8 MFS recurrences: Magnetic resonance and histological imaging. (**A**) The timeline of the patient’s medical history and treatment. Red boxes highlight the removal of the R7 and R8 MFS recurrences. (**B**) R7 localization: Upper third of the left shoulder before treatment, frontal section. (**C**) R8 localization: Thoracic cage, including the subscapular area, before treatment, axial section. (**D**) R7 histology: Neoplastic tissue with visible cellular pleomorphism (H&E staining, 100× magnification, scale bar = 100 µm). (**E**) R8 histology: Visibly more uniform neoplastic tissue (H&E staining, 100× magnification, scale bar = 100 µm).

Routine medical inspection revealed the presence of another recurrence around the original tumor site, with the core biopsy performed in May of 2024, suggesting a recurrence of MFS G3 (R8). On 25 June 2024 the removal of the recurrent (R8) soft tissue tumor in the left subscapular area was conducted (post-operation histology: MFS Grade 3). After the operation, the patient was dynamically monitored. Six months after the removal of the latest recurrence, new tissue growth was detected in the shoulder area, with possible ingrowth into the rib cage and the left scapula. The independent cell nodes in the lungs became larger and new nodes were detected, indicating potential metastatic spreading. Small nodes in the peribronchial area also hinted at the inflammatory/post-inflammatory processes. During the monitoring sessions from February to May of 2025, the metastases in the S1 part of the right lung and S2 part of the left lung became apparent on the CT. The summarized timeline of the patient’s medical history and treatment is presented in Figure 1A.

Taking into account the medical history of the patient, earlier (R7) and later (R8) tumor specimens, termed afterwards in the manuscript as the R7 and R8, present a unique case of fast-growing MFS recurrences, excised less than a year within one another and likely contributing to the metastatic dissemination. It is worthy of note that the patient received radiation therapy after the removal of the R2 recurrence in 2015 and chemotherapy (dacarbazine and doxorubicin) after the removal of the R4 recurrence in 2018. The long-term effects of this therapy on the tumor cannot be completely excluded. However, the patient did not receive chemotherapy or radiation treatment for 5 years prior to the excision of the R7 and R8 recurrences, which could have directly influenced the recurrent growth. The localization and histology of the R7 and R8 samples are summarized in Figure 1B–E.

The R7 and R8 samples (50–100 mg) were excised during the surgical operation, minced with a scalpel, and fixed in the 1X Fixation buffer (10x Genomics, Pleasanton, CA, USA). The tissue dissociation was performed according to the Tissue Fixation & Dissociation Protocol for Chromium Fixed RNA Profiling (10x Genomics, Pleasanton, CA, USA). The scRNA-seq libraries were prepared according to the Chromium Fixed RNA Protocol (10x Genomics, Pleasanton, CA, USA). The sequencing was performed on the Genolab M platform (GeneMind, Shenzhen, China) with 28 cycles for Read 1 and 90 cycles for Read 2.

Raw data from scRNA-seq was demultiplexed and aligned to the GRCh38-2020-A reference genome using the Cell Ranger v.7.1.0 (10x Genomics, Pleasanton, CA, USA). In total, 2039 cells were identified in the R7 sample with 11,548,336 total reads, 4701 reads per cell, and 1562 genes per cell, while the R8 contained 1986 cells (8,363,856 total reads, 3187 reads per cell, and 1154 genes per cell). Bioinformatic analysis was conducted using the Seurat package v.5.3.0 [18]. The preprocessing quality control (QC) workflow included the following parameters: cells with 300–10,000 detected genes, 500–20,000 unique molecular identifiers (UMIs), and mitochondrial transcript content below 10%. Potential doublets were identified and removed using the scDblFinder v.1.26.0 [19]. Data was normalized using the LogNormalize method and integrated with Harmony v.1.2.0 [20] to correct for batch effects. After quality control filtering, 1902 R7 cells and 1870 R8 cells were retained for analysis.

The MFS cells were clustered using the Leiden algorithm from the Seurat package v.5.3.0. Clustering robustness was assessed by testing a range of resolution parameters, yielding consistent cluster structures. Cluster annotation was based on cell ploidy and comparison of differentially expressed genes (DEG) against the canonical set of markers (Table S1). DEGs were identified using Seurat with the Wilcoxon Rank Sum test. Statistical thresholds for DEGs were at log fold change > 1 for comparisons between major cell types and at log fold change > 0.5 for subpopulation-level analyses within cell types, adjusted *p*-value < 0.05, corrected for multiple testing using the Benjamini–Hochberg method, and a minimal cell percentage of 0.25. The SCEVAN package v.1.0.3 was used to determine cell ploidy, with T cells being used as a reference. Cells labeled as aneuploid by the algorithm were considered tumor cells. Biological features of the cell clusters were determined by performing Gene Ontology (GO) and Hallmark pathway enrichment analyses on the DEG lists using the GSEA function from the clusterProfiler package v.4.16.0 [21].

**Figure 2:**
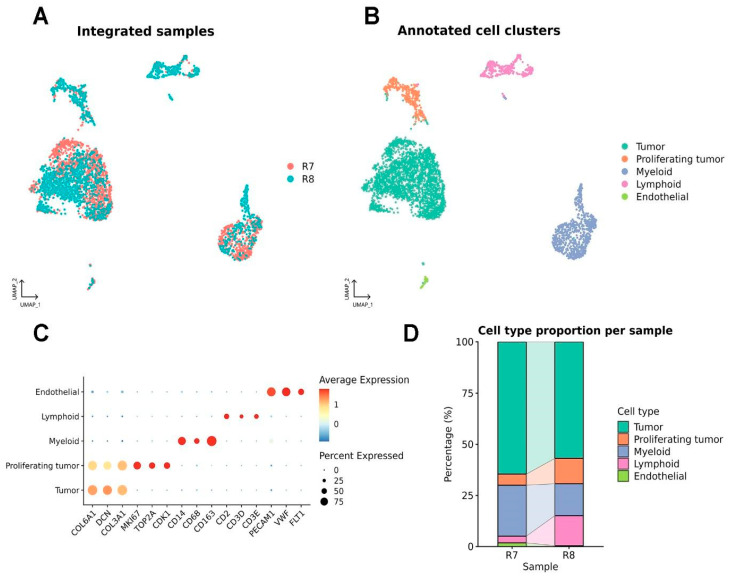
Annotation of the integrated cell populations of the R7 and R8 MFS recurrences. (**A**) UMAP visualization of the R7 and R8 cell alignment. (**B**) UMAP visualization of the annotated integrated R7 and R8 cell clusters. (**C**) Dot plot diagram showing the top 3 standard marker gene expressions across annotated R7 and R8 clusters. (**D**) Relative proportions of cell clusters in R7 and R8.

Five cell clusters were identified in the integrated R7 and R8 scRNA-seq dataset, including tumor cells (2293 cells, >85% aneuploidy), proliferating tumor cells (334 cells; >85% aneuploidy, *MKI67* and *TOP2A* expression), myeloid cells (765 cells; *CD14*, *CD68*, and *CD163* expression), lymphoid cells (337 cells; *CD2*, *CD3D*, and *CD3E* expression), and endothelial cells (43 cells; *PECAM1*, *VWF*, and *FLT1* expression) (Table S2; Figure 2A–C). R7 and R8 have a similar percentage of overall tumor cells (R7—70%; R8—69.3%). However, R8 displays a larger percentage of proliferating tumor cells (R7—5.4%; R8—12.4%), a smaller percentage of myeloid cells (R7—24.9%; R8—15.5%), and a significantly larger percentage of lymphoid cells (R7—3.3%; R8—14.7%). The percentage of endothelial cells is exceedingly low in both MFS recurrences (R7—1.8%; R8—0.4%) (Figure 2D).

**Figure 3:**
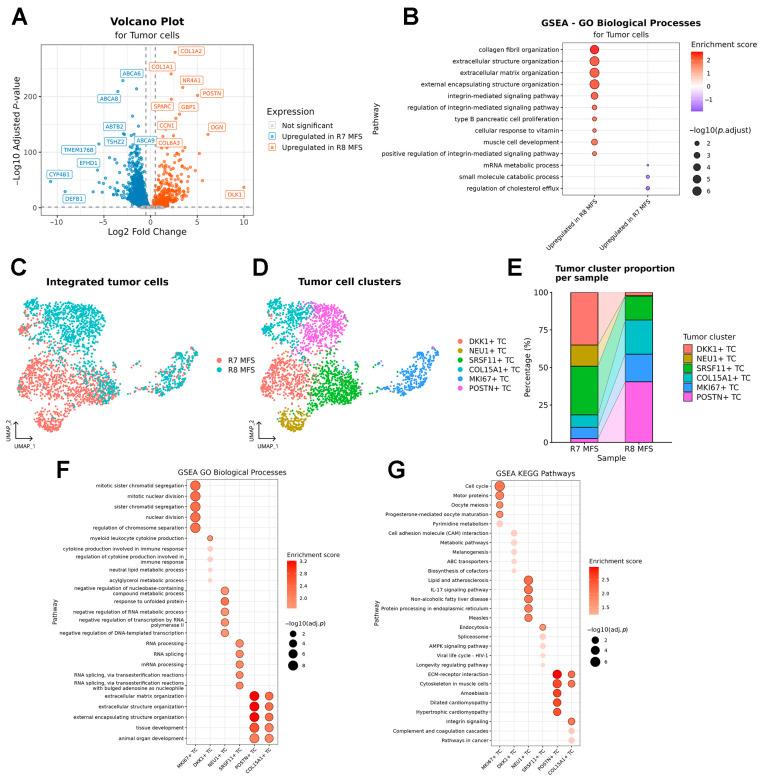
Characterization of tumor clusters (TCs) of the R7 and R8 MFS recurrences. (**A**) Volcano plot depicting the upregulation of genes in R7 and R8. (**B**) Dot plot of the top 10 GO biological processes enriched in R7 and R8. (**C**) UMAP visualization of the R7 and R8 tumor cell alignment. (**D**) UMAP visualization of the integrated MFS TC (R7 and R8). (**E**) Diagram showing the relative representation of tumor clusters in R7 and R8. (**F**,**G**) Dot plots of the top 5 upregulated GO biological processes and KEGG pathways enriched in the R7 and R8 TC.

Gene expression comparison of the R7 and R8 tumor cells (log2 fold change > 0.5, pct. > 0.25, adj. *p*-value < 0.05) revealed that the earlier R7 recurrence has increased expression of metabolic transporters and enzymes (*ABCA6*, *ABCA8*, *ABCA9*, *TMEM176B*, and *CYP4B1*), while the R8 recurrence has increased synthesis of ECM components (*COL1A1/2*, *COL6A3*, *POSTN*, *OGN*, and *LOXL3*) (Figure 3A,B). Tumor cells from the two recurrences were reclustered using the Leiden algorithm, and six tumor clusters (TCs) were identified: DKK+ TC (498 cells), NEU1+ TC (188 cells), SRSF11+ TC (639 cells), COL15A1+ TC (406 cells), MKI67+ TC (337 cells), and POSTN+ TC (559 cells) (Figure 3C,D). The DKK+ TC and NEU+ TC are specific to R7, the earlier MFS recurrence (Figure 3E). The DKK+ TC is enriched in immune system response (*DKK1*, *APP*, *CD24*, and *CD55*), stemness (*CD34* and *YJEFN3*), and GTPase activity (*ARHGAP1/12/21/17/27*) regulators. The NEU+ TC contains increased expression of lipid metabolism molecules (*NEU1*, *ABCA1*, *ABCA10*, *DDIT3*, and *ATF4*) and protein stress response molecules (the *DDIT3*, *ATF4*, *HSP*, and *DNAJ* family of genes) (Figure 3F,G; Table S3). The R7 and R8 recurrences share three TCs: SRSF11+ TC, COL15A1+ TC and MKI67+ TC. The SRSF11+ TC is enriched in RNA splicing and processing (*SNRNP70*, *SRSF2/5/11*, *RSRP1*, *LUC7L/7L3*, and *SRRM1/2*). The COL15A1+ TC displays increased expression of fibroblast and endothelial-specific collagens (*COL4A1/A2*, *COL15A1*, and *COL16A1*) and FGF/JUN/FOS signaling molecules (*FGF7*, *JUN/JUNB/JUND*, *FOS*, and *FOSB*). The MKI67+ TC is enriched in proliferative cells with a high expression of *MKI67*, *TOP2A*, and *BUB1B* (Figure 3F,G; Table S3). Additionally, R8, the later MFS recurrence, acquires the POSTN+ TC. This cluster contains markedly high expression of the ECM remodeling markers (*POSTN*, *COL1A1/A2*, *COL3A1*, and *NES*) typical for fibroblasts (Figure 3F,G; Table S3). Notably, the POSTN+ cluster demonstrates a high proportion of aneuploid cells (97.7%) based on SCEVAN analysis, comparable to other tumor clusters (>90%). This supports its classification as a malignant tumor population rather than stromal or cancer-associated fibroblast contamination (Figure S1).

**Figure 4:**
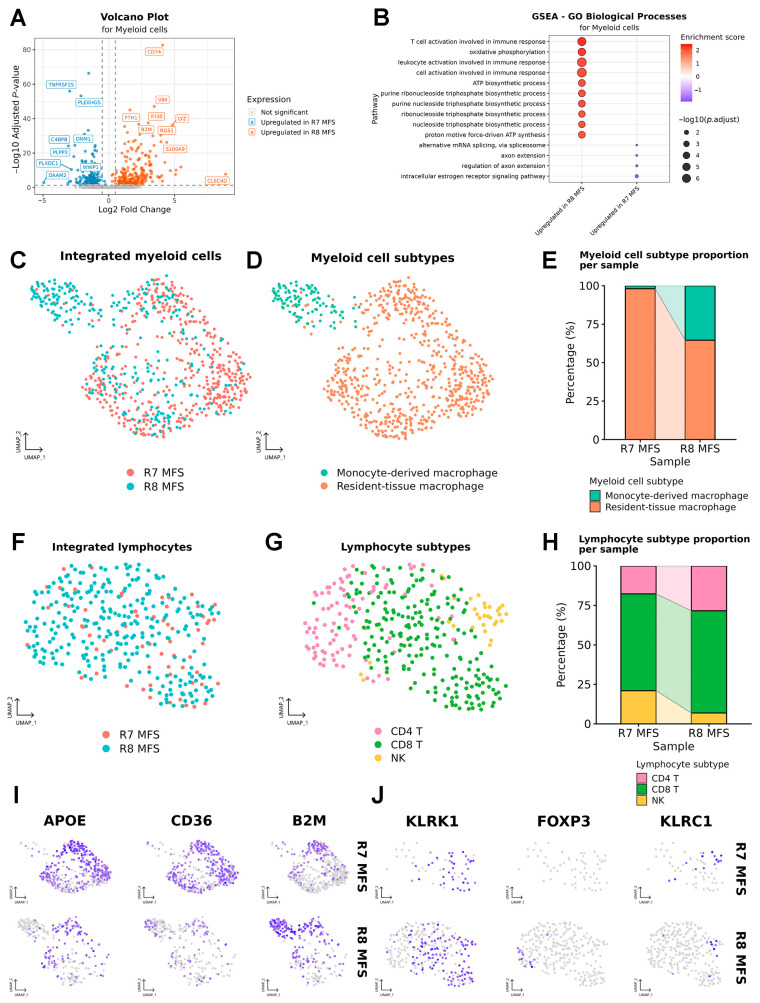
Characterization of the tumor microenvironment (TME) of the R7 and R8 MFS recurrences. (**A**) Volcano plot depicting the upregulation of genes in myeloid cells of R7and R8. (**B**) Dot plot of the top 10 GO biological processes enriched in myeloid cells of R7 and R8. (**C**) UMAP visualization of the R7 and R8 myeloid cell alignment. (**D**) UMAP visualization of the integrated MFS myeloid clusters (R7 and R8). (**E**) Diagram showing the relative representation of myeloid clusters in R7 and R8. (**F**) UMAP visualization of the R7 and R8 lymphoid cell alignment. (**G**) UMAP visualization of the integrated MFS lymphoid clusters (R7 and R8). (**H**) Diagram showing the relative representation of lymphoid clusters in R7 and R8. (**I**) Expression of the *APOE*, *CD36*, and *B2M* genes in myeloid cells of R7 and R8. (**J**) Relative expression of the *KLRK1*, *FOXP3*, and *KLRC1* genes in lymphoid cells of R7 and R8, with purple color indicating higher levels of expression.

The TME populations of R7 and R8 were explored through analysis of myeloid and lymphoid cells. The integrated myeloid cluster (Figure 4C) was reclustered using the Leiden algorithm, and the monocyte-derived macrophage cluster (112 cells) and the resident-tissue macrophage cluster (653 cells) were identified (Figure 4D,E). R8 displays an influx of monocyte-derived macrophages (R7 ~2%, 9 cells; R8 ~35%, 103 cells). The R7 myeloid cells display genes linked to the anti-inflammatory macrophage phenotype (*LYVE-1*, *LILRB5*, and *APOE*) and lipid metabolism genes (*PLPP3*, *APOE*, *CD36*, and *ABCA1*). The R8 myeloid cells are enriched in immune response and activation (*B2M*, *ZFP36*, *ZFP36L1*, and *ZFP36L2*) (Figure 4A,B,I; Table S4).

The integrated lymphoid cluster (Figure 4F) was reclustered, and the CD4+ T cell (89 cells), CD8+ T cell (216 cells), and NK cell clusters (32 cells) were identified (Figure 4G). R8 displays an increase in the overall number of lymphoid cells (R7 ~3%, 62 cells; R8 ~15%, 275 cells; Figure 2D), with CD4+ T cells and CD8+ T cells contributing to the cluster growth (R7 CD4+ T cells—11 cells, R8 CD4+ T cells—78; R7 CD8+ T cells—38 cells, R8 CD8+ T cells—178 cells) (Figure 4H). The Treg marker *FOXP3* has low levels of expression in the R7 and R8 CD4+ T cells, but in the case of R8 its expression is slightly higher. The R7 and R8 CD8+ T cells display cytotoxicity markers (*GZMH*, *KLRK1*, and *KLRG1*), while the NK cells express KLR genes responsible both for inhibitory and activating lectin-like receptors (*KLRF1* and *KLRC1/C3/C4*) (Table S5). In R8, an increase in the *KLRK1* expression in CD8+ T cells and NK cells is noted, while in R7 there is a slight increase in the expression of *KLRC1* in NK cells (Figure 4J).

## 3. Discussion

MFS is an understudied type of STS, with its challenging mode of growth resulting in higher rates of local recurrences and amputations than any other STS type [3]. The characterization of cell populations in MFS recurrences is required to find therapy solutions for this malignancy. In the present report, we use single-cell sequencing to investigate the tumor and TME populations of a recurrent MFS case with the subsequent development of metastases.

The analysis of MFS recurrence (R7 and R8) cells led to the identification of three common tumor clusters: SRSF11+ TC, COL15A1+ TC and MKI67+ TC. The SRSF11+ TC is a close match to the SNRNP70+ TC previously identified for the primary MFS [11], with analogous enrichment in splicing factors (*SNRNP70*, *RSRP1*, and *LUC7L*). The MKI67+ TC is similar to the highly proliferative cell group also previously seen in primary MFS [11]. The COL15A1+ TC may correspond to the previously described COL1A2+ TC in primary MFS [11], although the COL15A1+ TC displays an additional enrichment in the FGF-induced Jun/Fos signaling (*FGF7* and *JUN/FOS*) and collagens shared by fibroblasts and vascular smooth muscle cells (*COL4A1/15A1/16A1*). The presence of tumor cells with enrichment in proliferation and RNA editing and splicing may hypothetically be the common denominator for the primary and recurrent MFS. While the role of this cluster is unclear at present, splicing as a biological process may aid MFS tumor cell expansion and adaptation to the changing conditions. Previously, the overexpression of the serine–arginine-rich splicing factors (SRSFs) has been linked to aggressiveness and metastasis in NSCLC and colorectal cancer [22,23]. In osteosarcoma, the U1 small nuclear ribonucleoprotein 70 kDa (snRNP70) promotes metastasis through regulation of the *CD55* transcript splicing [24]. The RNA-binding protein 39 (RBM39) is linked to Ewing sarcoma progression, and the RBM39 degrader is currently undergoing clinical trials [25]. Analysis of a larger patient cohort is still needed to verify the expression of these splicing genes in the primary and recurrent MFS, as well as to define the potential role of the splicing-gene signature in MFS aggressiveness, local recurrence, and metastasis.

The DKK+ TC and the NEU+ TC are observed only in the earlier R7 recurrence. Both clusters express genes (*DKK1*, *APP*, *CD24*, and *GRN*) that have been suggested to play immunosuppressive roles [26,27,28]. The expression of these immunomodulary markers has been detected previously in tumor clusters of pleomorphic sarcomas with the immune-hot phenotype, pleomorphic rhabdomyosarcoma and UPS [29,30]. The NEU+ TC is enriched in the genes of lipid metabolism, a metabolic process that has also previously been linked to immunosuppression in various cancers [31]. This corresponds with the R7 recurrence having a significantly smaller lymphoid cluster than R8 (Figure 2D), and its myeloid cells bearing genes linked to anti-inflammatory functions (*APOE*, *LYVE-1*, and *LILRB5*) (Figure 4I) [32,33,34]. Additionally, the expression of *DDIT3*, *ATF4*, and *HSPA/DNAJ* in the NEU+ TC might be linked to the adaptive response to cell stress [35,36,37]. A similar gene signature was observed in one of the tumor populations in a recent single-cell study of UPS [30]. It can be hypothesized that these adaptive qualities help the R7 recurrence maintain steady growth without triggering high immune response, although analysis of a larger cohort of MFS recurrences is necessary to verify this.

In the later recurrence (R8), the DKK+ TC and NEU+ TC are absent, and the POSTN+ TC is present instead. The presence of the POSTN+ TC in R8 correlates with the more visibly uniform cell morphology, compared to R7 (Figure 1D,E). According to the patient’s medical history, the R8 recurrence was followed by the development of lung metastases within less than a year (Figure 1A). High expression of nestin, collagens, and other cytoskeletal genes in the R8 POSTN+ TC suggests a role in cell migration, which may have contributed to the subsequent metastatic process. However, this is a hypothesis, since the metastatic MFS tissue was not analyzed in this case report. Additionally, verification of this statement on a larger cohort is required. The R8 macrophages demonstrate signs of immune response activation. A corresponding influx of lymphoid cells (Figure 2D) is accompanied by an increase in *KLRK1* expression in CD8+ T cells, a slight increase in the expression of the Tregs marker *FOXP3* in CD4+ T cells, and a slight decrease in the *KLRC1* expression in NK cells (Figure 4J). Overall, this shift points to a pro-inflammatory TME.

In summary, the earlier R7 recurrence contains more diverse tumor populations involved in immunomodulation, lipid metabolism, cell stress response, RNA splicing, ECM remodeling and proliferation. The later R8 recurrence is mainly enriched in ECM remodeling, proliferation and RNA splicing. The TME from R7 to R8 experiences a shift towards the pro-inflammatory type.

This study has its limitations. First, while the R7 and R8 recurrences have not received chemotherapy treatment prior to their excision, the patient’s medical history reveals radiotherapy and chemotherapy treatment 5 years prior. Analysis of MFS recurrences without any prior treatment interference is necessary to verify the case report’s findings. Second, the transcriptomic markers of the TC that R7 and R8 share with the primary MFS have been verified on a previously published scRNA-seq dataset [11], but the novel MFS TC have only been documented on one patient. A confirmation of this data on a larger patient cohort is required. Additionally, the limited availability of primary MFS scRNA-seq datasets restricts more systematic cross-cohort comparisons. Third, the protein level verification of our transcriptomic findings, including the expression of *DKK1*, *SRSF11*, and *POSTN* in the recurrent MFS TC, is necessary. Fourth, the difference in sequencing depth between R7 (4701 reads/cell) and R8 (3187 reads/cell) should be considered when interpreting the observed differences in functional diversity. However, both values fall within the expected range for the Chromium Fixed RNA Profiling chemistry, and the cluster-defining markers of the R7- and R8-specific TCs are robustly expressed genes unlikely to be lost due to the technical dropout. LogNormalize normalization and Harmony integration were applied to mitigate potential biases introduced by variable sequencing depth. Fifth, the limited number of non-tumor cells, partly reflecting the multiplexed pooling strategy and the biological composition of the specimens, constrains the depth of the TME analysis.

## 4. Conclusions

This is the first study of the consequent MFS recurrences of one patient at the single-cell level. The earlier MFS recurrence contains unique TCs with enrichment for immunomodulatory markers (*DKK1*, *APP*, *CD24*, and *GRN*) and genes involved in lipid metabolism and cell stress modulation (*DDIT3*, *ATF4*, *ABCA1*, *ABCA10*, and *NEU1*). The later MFS recurrence, which was excised less than a year before the detection of lung metastases, is less functionally diverse, enriched in fibroblast-typical markers (*POSTN*, *NES*, and *COL1A1/A2*), and displays a shift to pro-inflammatory TME. MFS recurrences share two tumor clusters with enrichment in proliferation (*MKI67*, *TOP2A*, and *BUB1B*) and mRNA splicing and processing (*SNRNP70*, *SRSF2/5/11*, *RSRP1*, *LUC7L/7L3*, and *SRRM1/2*) that correspond with the previously described primary MFS tumor populations. Since this is a case report, the tumor and TME cell signatures described here require verification on a larger cohort of MFS patients.

## Data Availability

The datasets generated during the current study are not publicly available due to ethical and privacy restrictions related to patient-derived data and the terms of informed consent but are available from the corresponding author on reasonable request and subject to institutional approval.

## Supplementary Materials

The following supporting information can be downloaded at: https://www.mdpi.com/article/10.3390/ijms27146229/s1.
