# Supplementary figures and images for: Evolution of Recurrent Myxofibrosarcoma of the Thoracic Wall at Single-Cell Resolution: A Case Report
Source: Int J Mol Sci. 2026 Jul 13;27(14):6229. doi: 10.3390/ijms27146229 (PMC13410272; doi:10.3390/ijms27146229)

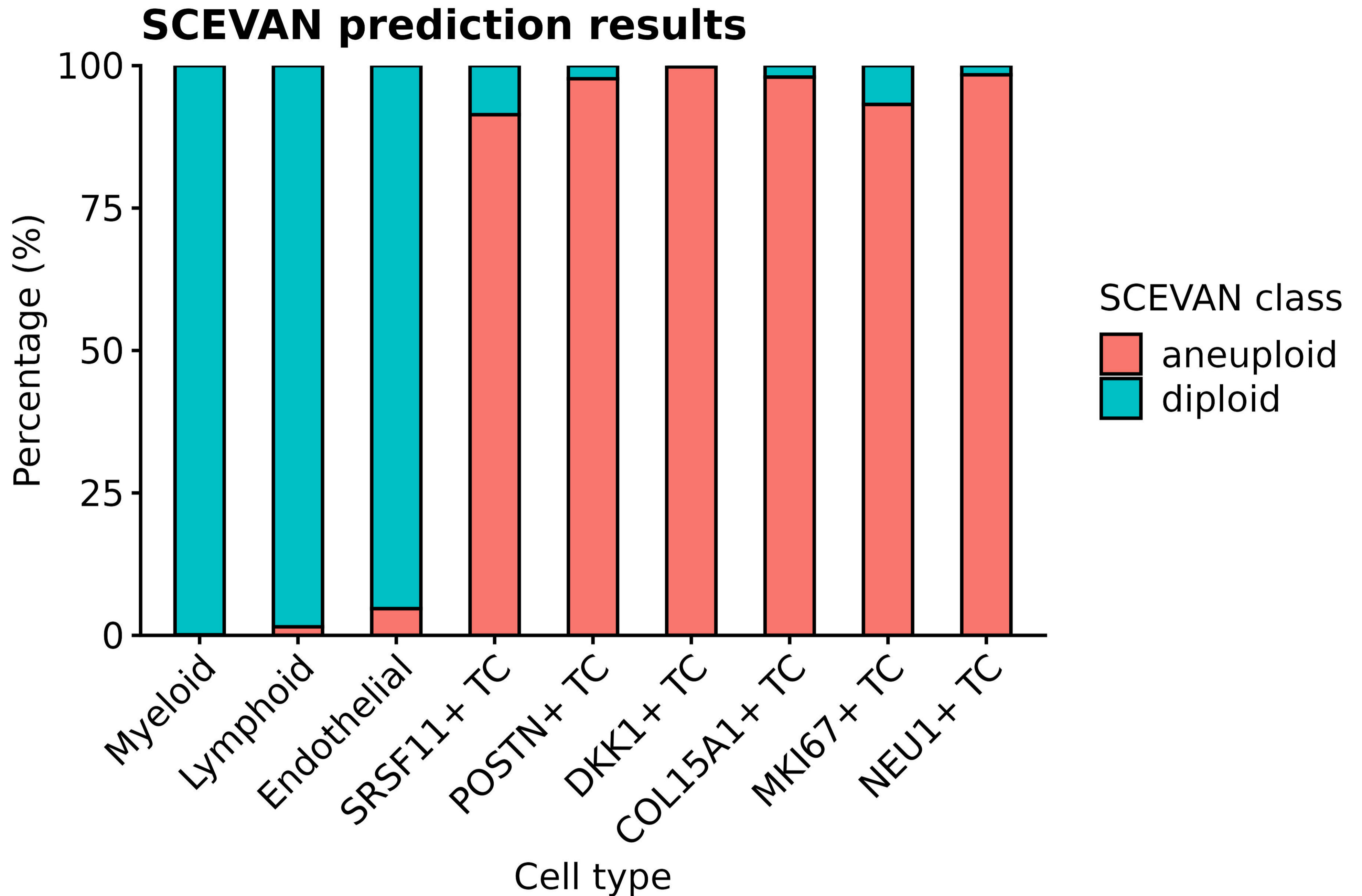

**Figure S1.** The percentage of diploid and aneuploid cells in R7 and R8 cell clusters.

Supplement: Supplementary file 1 [file ijms-27-06229-s001.zip › Supplementary Figures.pdf]
